# The relationship between fear of recurrence and depression in patients with cancer: The role of invasive rumination and catastrophizing

**DOI:** 10.3389/fpsyt.2022.920315

**Published:** 2022-09-20

**Authors:** Lijuan Quan, Xinxin Wang, Wei Lu, Xintong Zhao, Jialei Sun, Qingsong Sang

**Affiliations:** ^1^School of Educational Science, Anhui Normal University, Wuhu, Anhui, China; ^2^Foreign Language School of Ma’anshan No. 2 Middle School, Ma’anshan, China

**Keywords:** fear of recurrence, invasive rumination, catastrophizing, depression, cancer

## Abstract

**Objective:**

To examine the relationship between fear of recurrence and depression in patients with cancer.

**Materials and methods:**

Two hundred and fifty-nine participants completed self-report questionnaires, including the Fear of Progression Questionnaire-Short Form, Rumination Inventory, Cognitive Emotion Regulation Questionnaire (Chinese version), and Center for Epidemiological Studies Depression Scale.

**Results:**

Fear of recurrence in patients with cancer was moderate, and the level of depression was significantly higher than that in the normal population. Fear of recurrence, invasive rumination, catastrophizing, and depression in patients with cancer were significantly positively correlated. The level of fear of recurrence was a significant positive predictor of the level of depression. Invasive rumination played a partial mediating role between fear of recurrence and depression; that is, fear of recurrence directly affected depression, and fear of recurrence indirectly affected depression through invasive rumination. Catastrophizing played a moderating role in the mediation model, in which fear of recurrence affected depression through invasive rumination.

**Conclusion:**

Invasive rumination plays a mediating role between fear of recurrence and depression in patients with cancer. Catastrophizing moderates the relationship between fear of recurrence and depression as well as the relationship between invasive rumination and depression.

## Introduction

Cancer and its treatment can cause a whole host of painful and physically traumatic symptoms. Less well understood, perhaps, is the immense psychological trauma that patients often experience throughout the processes of diagnosis, treatment, and recovery. Patients with cancer often display a series of negative psychological responses, such as fear of recurrence, anxiety, depression, and insomnia (1–3). Among these, the most common is depression, which has a serious impact on patients’ quality of life, treatment, rehabilitation, and survival (4–7). Furthermore, depression affects the treatment of patients with cancer, leading to various physical symptoms and can even cause suicidal thoughts (8–10). Therefore, the study of depression in patients with cancer plays an essential role in improving their physical and mental health.

Fear of recurrence is also a common negative emotional response among patients with cancer. It is a continuous process that occurs at all stages of cancer (11, 12). The fear of recurrence can transition from normal discomfort to clinical symptoms. Generally speaking, low levels of fear of recurrence can be considered a normal emotional response to cancer and can remind patients to pay attention to the disease recurrence and help them improve their overall quality of life (13, 14). However, when severe enough to become a significant clinical problem, it can lead to irrational worry, anxiety, frequent intrusive thinking, negative coping styles, excessive grief, functional impairment, and difficulty in making future plans (11).

Predictably, there are links between the fear of recurrence and depression. Some studies have found that fear of recurrence can lead to increased anxiety and depression. These negative emotions affect patients’ psychology, which in turn can reduce the effectiveness of cancer drugs or cause adverse drug reactions. This further affects patients’ physical and mental health (15). In addition, a study has found that when patients with cancer experience fear of recurrence, they tend to focus too much on their own physical condition and may become excessively preoccupied with health and physical changes. These patients may interpret symptoms, such as pain and depression, as signs of disease aggravation and are more likely to develop depression (16).

According to the theory of cognitive appraisal (17), when a person is in a stressful situation, (s)he needs to make cognitive assessments of the external stimuli and respond accordingly in different ways. For traumatic events that are highly destructive, such as cancer, it is difficult for individuals concerned about recurrence to make a positive assessment within a short period. Instead, they react negatively to traumatic events (18). Invasive rumination is a negative coping style commonly adopted by individuals after experiencing traumatic events such as cancer (19). It is a maladaptive thought pattern that is used by individuals to regulate negative emotions and is an independent component of depressive rumination. Invasive rumination is often associated with high levels of fear of recurrence in patients with cancer. Therefore, Hypothesis 1 proposes that invasive rumination plays a mediating role between the fear of recurrence and depression in patients with cancer.

According to the risk enhancement model (20), one risk factor enhances the role of the other. Different cognitive emotion regulation strategies have different effects on depression levels. Catastrophizing is another maladaptive strategy of cognitive emotion regulation (21). It is a way that when individuals are faced with negative life events, they use their worst thoughts for emotional regulation. When cancer is diagnosed, patients typically experience more negative emotions (22). In attempting to regulate their emotional state, patients may adopt irrational, negative attitudes and negative coping styles, which inevitably make their negative emotional state more serious. Patients with cancer who engage in catastrophizing are more likely to experience enhanced levels of invasive rumination and depression. Therefore, Hypothesis 2 is proposed: catastrophizing plays a significant moderating role in the mediation model of the impact of fear of recurrence on depression.

**FIGURE 1:**
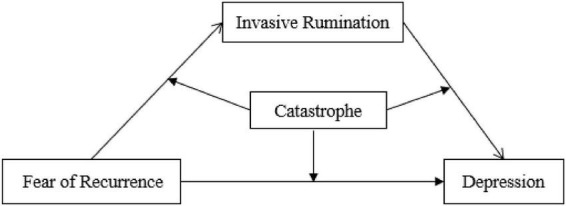
Hypothetical model of the study.

The hypothesis model of this study is shown in Figure 1.

## Materials and methods

### Participants

This study was approved by the Research Ethics Committee of Anhui Normal University. Paper questionnaires were distributed to the oncology department of hospitals in Wuhu, Hefei, and Bozhou City of Anhui Province, China. There were 259 patients with cancer, including 113 men (43.6%) and 146 women (56.4%). Among these participants, 42 people (16.2%) were aged 18–44, 154 (59.5%) were aged 45–59, and 63 people (24.3%) were aged over 60, and they effectively completed the questionnaires (Table 1). The inclusion criteria were as follows: (1) medically diagnosed cancer, (2) mentally clear and aware of their conditions, (3) able to communicate normally, and (4) signed informed consent. The exclusion criteria were: (1) people with mental or cognitive disorders and (2) people who knew nothing about their own conditions.

**TABLE 1:** Basic information of the participants in a valid questionnaire.

Demographic variables	Standard	Number of samples	Percentage (%)
Gender	(1) Male	113	43.6
	(2) Female	146	56.4
Age	(1) 18–44 Years old	42	16.2
	(2) 45–59 Years old	154	59.5
	(3) More than 60	63	24.3
Marital status	(1) Widowed	19	7.3
	(2) Divorced	21	8.1
	(3) Married	219	84.6
Number of children	(1) None or one child	137	52.9
	(2) Two and more children	122	47.1
Age of children	(1) <18	33	12.7
	(2) 18–35	175	67.6
	(3) >35	51	19.7

### Measures

#### Fear of progression questionnaire-short form

The Short Form of the Fear of Progression Questionnaire prepared by Mehnert et al. (23) and revised by Wu et al. (24) was used to measure fear of recurrence in patients with cancer. The scale is divided into two subscales, physiological health, and social and family, with a total of 12 questions. The questionnaire measured the level of fear of recurrence by the score (12–23, 24–36, and 36–60 for low, medium, and high levels of fear of recurrence, respectively) (23). In this study, Cronbach’s α coefficient of the questionnaire was 0.89, indicating good internal consistency.

#### Rumination inventory

Levels of invasive rumination in patients with cancer were measured using the Invasive Rumination Dimension of the Rumination Inventory. This questionnaire was modified by Zhou et al. (25) based on Cann et al. (26) event-related rumination inventory, which was divided into two sub-questionnaires: invasive rumination and active rumination, with a total of 20 items. In this study, the Cronbach’s α coefficient for the invasive rumination sub-questionnaire was 0.91, indicating good internal consistency.

#### The cognitive emotion regulation questionnaire-Chinese version

The catastrophizing dimension of the cognitive emotion regulation questionnaire-Chinese version (CERQ-C) was used to measure catastrophizing as an emotion regulation strategy in individuals (21, 27). In this study, Cronbach’s α coefficient of the catastrophizing sub-questionnaire was 0.81, indicating good internal consistency. The questionnaire consisted of 36 items, including nine dimensions: self-blaming, acceptance, rumination, positive re-attention, planning, positive reappraisal, downward comparison, catastrophizing, and blaming others. A Likert 5-point scale was used to score from “never” to “always,” using “1” to “5,” respectively.

#### Center for epidemiologic studies depression scale

Center for epidemiologic studies depression scale (CES-D) was used to assess depression levels. The CES-D was originally prepared by Radloff (28) and later revised by Chen et al. (29). Researchers such as Zhang et al. (30) established the standard of depression score of the general population (13.24 ± 10.33 points). The scale was mainly used to measure the degree of depression in the participants in the last week of this study, with a total of 20 items, including four dimensions of depression, positive emotion, physical symptoms, and interpersonal relationships. In this study, Cronbach’s α coefficient of the scale was 0.91, indicating good internal consistency.

### Procedures

With the consent of the hospital, the researcher was led into the ward by a nurse and shared questionnaires with patients who were not under treatment and were relatively comfortable. After ensuring that the participants understood the manner and requirements of the answers, they were asked to sign an informed consent form and then complete the questionnaire. Upon completion of the responses, the staff recovered the questionnaire on-site. After the questionnaires were collected, the researchers played a relaxing and pleasant video to eliminate the negative experience of answering the questionnaire and informed the participants that psychological counseling services were available.

### Data analysis strategies

Statistical software SPSS 24.0 was used for descriptive statistics, difference tests, correlation analysis, etc. AMOS 22.0 was used for the validity test of the questionnaire, and SPSS macro program PROCESS developed by Hayes was used for the model test.

## Results

### Common method bias test

The Harman’s single-factor test was used for the common method bias test. Eight common factors with eigenvalues greater than 1 were identified in this study, and the variation rate explained by the first factor was 35.72%, which was less than the standard of 40%. Therefore, it can be considered that there was no serious common method bias in this study.

### Descriptive statistics of each variable

A series of descriptive statistical analyses were conducted on fear of recurrence, invasive rumination, catastrophizing, and depression in patients with cancer, and the results are shown in the table below.

As shown in Table 2, the mean scores of the two dimensions of fear of recurrence in patients with cancer, namely, physical health, and social and family, are similar. The mean score for fear of recurrence was 35.92, which was in the middle range (24–36). The mean score and standard deviation of depression were 28.03 and 11.68, respectively, which were significantly higher than those of the general population (13.24 ± 10.33, *t* = 20.38, *P* < 0.001).

**TABLE 2:** Descriptive statistics of fear of recurrence, invasive rumination, catastrophizing, and depression.

	*M*	*SD*
Physical health	18.37	5.32
Society and family	17.55	5.17
Fear of recurrence	35.92	9.75
Invasive rumination	15.38	6.44
Catastrophizing	7.38	3.46
Depressed mood	10.24	5.98
Positive emotion	6.81	2.82
Somatic symptoms	9.11	3.73
Interpersonal relationships	1.87	1.71
Depression	28.03	11.68

### Correlation analysis of each variables

To explore the relationship between fear of recurrence and depression in patients with cancer, correlation analysis tests were conducted on fear of recurrence, invasive rumination, catastrophizing, and depression (Table 3).

**TABLE 3:** Correlation coefficients of fear of recurrence, invasive rumination, catastrophizing, and depression.

	1	2	3	4	5	6	7	8	9	10
1. Physical health	1									
2. Society and family	0.73**	1								
3. Fear of recurrence	0.93**	0.93**	1							
4. Invasive rumination	0.62**	0.65**	0.68**	1						
5. Catastrophizing	0.56**	0.62**	0.63**	0.63**	1					
6. Depressed mood	0.64**	0.68**	0.70**	0.68**	0.64**	1				
7. Positive emotion	0.28**	0.32**	0.32**	0.32**	0.32**	0.40**	1			
8. Somatic symptoms	0.52**	0.55**	0.57**	0.62**	0.52**	0.77**	0.26**	1		
9. Interpersonal relationships	0.40**	0.47**	0.47**	0.46**	0.45**	0.63**	0.27**	0.56**	1	
10. Depression	0.62**	0.66**	0.69**	0.69**	0.64**	0.95**	0.57**	0.86**	0.71**	1

**p < 0.01.

### Regression analysis of depression and fear of recurrence

A regression analysis was conducted with fear of recurrence as the predictive variable and depression as the outcome variable. Referring to the research method of Wen and Ye (31), demographic variables, such as gender, age, family economic status, marital status, number of children, and age of children, were set as dummy variables and controlled in the first layer of regression analysis. After controlling for demographic variables, fear of recurrence was a significant positive predictor of depression in patients with cancer (β = 0.80, *p* < 0.001). The results are presented in Table 4.

**TABLE 4:** Regression analysis of depression to fear of recurrence.

Antecedent variables	Unstandardized coefficients		Standardized coefficients		*R* ^2^
	*B*	SD	*B*	*t*	
Female	−1.13	1.07	−0.05	−1.05	
Age 18−44	−1.73	1.77	−0.55	−0.98	
Age above 60	−5.67	1.76	−0.21	−3.23**	
Monthly income less than 1,000 yuan	3.03	1.61	0.11	1.88	
Monthly income between 3,000 and 5,000 yuan	2.19	1.58	0.08	1.38	
Monthly income between 5,000 and 7,000 yuan	0.59	1.84	0.02	0.32	
Monthly income between 7,000 and 9,000 yuan	3.01	2.02	0.08	1.49	
Monthly income above 9,000 yuan	1.05	1.95	0.03	0.54	
Widowed	0.97	2.13	0.22	0.46	
Divorced	3.05	1.96	0.07	1.55	
Two or more children	−0.38	1.11	−0.02	−0.34	
Children under the age of 18	−3.97	1.96	−0.11	−2.03*	
Children over the age of 35	3.95	1.95	0.14	2.03*	0.12
Fear of recurrence	0.80	0.06	0.67	14.52***	0.53

*p < 0.05, **p < 0.01, and ***p < 0.001.

### Mediating role of invasive rumination

Fear of recurrence, depression, and invasive rumination were set as the independent, dependent, and intermediate variables, respectively. The SPSS macro program PROCESS software compiled by Hayes (32) was used to test the mediating effect using the three-step test method. The results are presented in Table 5.

**TABLE 5:** Analysis of mediating role of invasive rumination in fear of recurrence and depression.

	Equation 1: Invasive rumination			Equation 2: Depression		
	*B*	SE	*t*	β	SE	*t*
Fear of recurrence	0.46	0.03	15.20***	0.45	0.07	6.57***
Invasive rumination				0.80	0.10	7.72***
R^2^	0.50			0.59		
F	35.26***			44.86***		

***p < 0.001.

It was concluded that fear of recurrence was a significant positive predictor of invasive rumination (β = 0.46, *P* < 0.001). Fear of recurrence positively predicted depression. The positive effect of fear of recurrence on depression was slightly reduced after the addition of invasive rumination, but it remained significant (β = 0.45, *P* < 0.001). Furthermore, invasive rumination was a positive predictor of depression (β = 0.80, *P* < 0.001). Accordingly, invasive rumination partially mediated the relationship between fear of recurrence and depression, accounting for 44.82% of the total effect. The variance of the dependent variable, depression, explained by the mediating variable, invasive rumination, was 30.59%.

### Moderating role of catastrophizing emotion regulation strategy

Fear of recurrence, depression, invasive rumination, and catastrophizing emotion regulation strategy were set as the predictive, dependent, mediating, and moderating variables, respectively. SPSS macro program PROCESS software was used to test the moderated mediating effect. The results are presented in Table 6.

**TABLE 6:** Moderating role of catastrophizing emotion regulation strategy on fear of recurrence, invasive rumination, and depression.

	Equation 1: Depression			Equation 2: Invasive rumination			Equation 3: Depression		
	β	SE	*t*	β	SE	*T*	β	SE	*t*
Fear of recurrence	0.36	0.07	5.20***	0.33	0.04	8.95***	0.39	0.07	5.50***
Catastrophizing	0.87	0.19	4.71***	0.60	0.10	5.74***	0.90	0.18	4.89***
Fear of recurrence × catastrophizing	0.03	0.01	2.64**	−0.002	0.01	−0.20			
Invasive rumination							0.59	0.11	5.56***
Invasive rumination × catastrophizing							0.06	0.02	3.29**
*R* ^2^	0.63			0.56			0.63		
*F*	41.88***			34.81***			42.90***		

**p < 0.01; ***p < 0.001.

**FIGURE 2:**
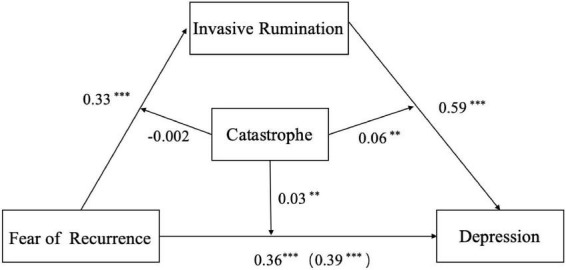
Catastrophizing moderated the mediation model of fear of recurrence to invasive rumination to depression: Statistical diagram. ^**^p < 0.01; ^***^p < 0.001.

In Equation 1, fear of recurrence was a significant positive predictor of depression (β = 0.36, *P* < 0.001), and the interaction between fear of recurrence and catastrophizing emotion regulation significantly predicted depression (β = 0.36, *P* < 0.001). Thus, catastrophizing can moderate the relationship between fear of recurrence and depression. In Equation 2, fear of recurrence had a significant positive predictive effect on invasive rumination (β = 0.33, *P* < 0.001), and the interaction between fear of recurrence and catastrophizing had no significant predictive effect on invasive rumination (β = −0.002, *P* > 0.05), indicating that catastrophizing cannot moderate the relationship between fear of recurrence and invasive rumination. In Equation 3, fear of recurrence was a significantly positive predictor of depression (β = 0.39, *p* < 0.001), invasive rumination had a significant positive predictive effect on depression (β = 0.59, *P* < 0.001), and the interaction between invasive rumination and catastrophizing also significantly predicted depression (β = 0.06, *P* < 0.01), indicating that catastrophizing can moderate the relationship between invasive rumination and depression. Therefore, invasive rumination plays a mediating role in the relationship between fear of recurrence and depression, and catastrophizing emotion regulation strategy plays a moderating role between fear of recurrence and depression, as well as between invasive rumination and depression (Figure 2).

**FIGURE 3:**
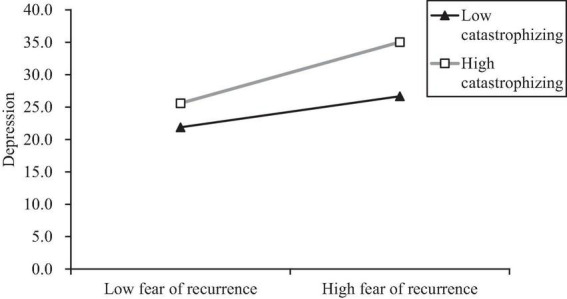
Moderating role of catastrophizing on fear of recurrence and depression.

To further explore the nature of the interaction effects between fear of recurrence and catastrophizing, catastrophizing was divided into high and low groups with plus or minus one standard deviations, and the simple effect test was used to investigate the effect of fear of recurrence on depression in different degrees of catastrophizing emotion regulation strategy. The results showed that fear of recurrence had a significant predictive effect on depression at low catastrophizing levels (β = 0.25, *t* = 3.03, *P* < 0.01), and a more significant predictive effect at high catastrophizing levels (β = 0.48, *t* = 5.63, *P* < 0.001). Therefore, catastrophizing has a significant effect on emotional regulation; when catastrophizing is high, fear of recurrence has a greater impact on depression than when it is low (Figure 3).

**FIGURE 4:**
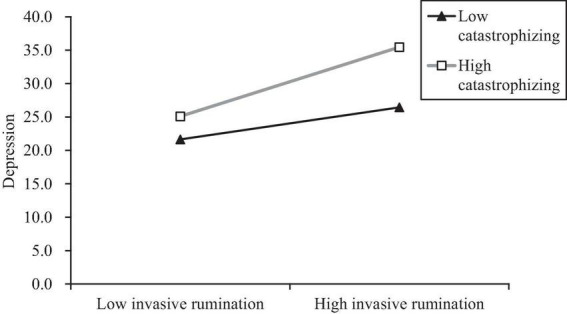
Moderating role of catastrophizing on invasive rumination and depression.

To further explore the nature of the interaction effects between invasive rumination and catastrophizing, catastrophizing was divided into high and low groups with plus or minus one standard deviations, and the simple effect test was used to investigate the impact of invasive rumination on depression in different degrees of catastrophizing emotion regulation strategy. The results showed that, invasive rumination had a significant predictive effect on depression (β = 0.37, *t* = 2.81, *p* < 0.01) at low catastrophizing levels, and a more significant predictive effect at high catastrophizing levels (β = 0.80, *t* = 6.92, *p* < 0.001). Therefore, catastrophizing has a significant moderating effect; that is, when catastrophizing is at a high level, invasive rumination has a greater impact on depression than when it is at a low level (Figure 4).

## Discussion

### Current situation analysis of fear of recurrence and depression

In this study, the average fear of recurrence score of patients with cancer reached the middle level, close to the high level, indicating that the levels of fear of recurrence cannot be ignored. This is consistent with the findings of previous studies (33, 34). In the fear of recurrence, the physical health dimension score was higher than that of the social family dimension, which confirmed the results of previous studies (35). This is related to the long treatment cycle and high recurrence rate of cancer. Moreover, in the treatment process, patients must endure the pain associated with radiotherapy and chemotherapy, accompanied by physical symptoms. Thus, patients are more fearful that the disease will recur and that they will have to endure the pain again. Therefore, in clinical practice, medical staff should not only pay attention to the recurrence of cancer from a medical perspective but also be concerned about the levels of fear of recurrence. They should provide timely guidance and clinical intervention to encourage patients to become more actively involved in their own treatment and improve their physical and mental health.

Compared with the standard depression level of the general population established by Zhang et al. (30), the depression score of patients with cancer in this study is significantly higher, which is consistent with the results of previous studies (2, 4). This is because patients with cancer, compared to the general population, have to endure not only the physical pain caused by a long cycle of chemotherapy and treatment but also the negative emotions and experiences brought by the treatment process. When they are unable to deal with these negative emotions, their depressed mood may become more pronounced.

### Analysis of mediating role of invasive rumination

There was a significant positive correlation between fear of recurrence and depression in patients with cancer, indicating that the higher the fear of recurrence, the higher the level of depression. This is consistent with previous studies (34, 35). Given these findings, medical staff in clinical practice should pay close attention to observe the psychological changes in patients with cancer. Medical staff can then identify depression in a timely manner and provide positive guidance to avoid the occurrence or aggravation of the depressed mood.

According to the results of the mediating effect analysis, invasive rumination plays a mediating role in the relationship between fear of recurrence and depression. Fear of recurrence can not only directly predict the level of depression in patients but also predict the level of depression through the mediating role of invasive rumination. This is in line with the theory of cognitive appraisal (17). For traumatic events that are highly destructive, such as cancer, patients who are afraid of recurrence find it difficult to actively understand and digest them in a short time, so they instinctively adopt negative coping styles such as invasive rumination to deal with such events (18, 36). In addition, according to the affective cognition model, people have a stable perception of the world and themselves before traumatic events, such as cancer, occur. However, when cancer develops, it challenges previous beliefs. At this time, individuals’ sense of helplessness and incompetency are hit. There is a sharp contrast between perceptions before and after the diagnosis of cancer. When individuals cannot accept this contrast, they adopt a series of negative coping styles, such as invasive rumination (37). Depression can occur when individuals repeatedly recall painful experiences of cancer.

It can be seen from the characteristics of fear of recurrence that it is often accompanied by frequent invasive thinking, during which patients with cancer cannot help recall the painful experience of cancer. As a negative coping style, invasive rumination undoubtedly increases the psychological burden on patients and leads to a depressed mood (38).

This finding has clinical value, which helps us recognize the negative role of invasive rumination in the relationship between fear of recurrence and depression and help patients avoid or alleviate the negative thinking of invasive rumination to support them in leading healthier lives.

### Analysis of the moderating role of catastrophizing

Analysis of the moderating role of catastrophizing showed that it moderated the relationship between fear of recurrence and depression in patients with cancer. With an increase in catastrophizing levels, the positive correlation between fear of cancer recurrence and depression was enhanced. Catastrophizing also moderated the relationship between invasive rumination and depression. With an increase in catastrophizing levels, the positive correlation between invasive rumination and depression strengthened. Thus, Hypothesis 2 was verified.

There was a positive correlation between catastrophizing and depression, indicating that the higher the level of catastrophizing, the higher the level of depression. This is consistent with the results of previous studies (39). Individuals unconsciously experience negative psychological reactions after suffering from cancer. They are prone to catastrophic thoughts after a cancer diagnosis and believe that cancer is terrible. This negative emotion-regulation strategy can exacerbate depression.

Catastrophizing moderated the relationship between fear of recurrence and depression. The effect of fear of recurrence on depression was greater at high catastrophizing levels than at low catastrophizing levels. This can be explained by the risk enhancement model. It could also be that patients with cancer attempt to eliminate or regulate these negative emotions after getting sick (40). However, under the torture of the illness, some patients cannot find an effective positive emotion regulation strategy in a short period. As a result, they adopt a common negative regulation strategy of catastrophizing (41, 42). When individuals have catastrophizing thoughts, thereby enhancing their level of fear of cancer recurrence, the level of depression increases correspondingly. In other words, catastrophizing intensifies the relationship between fear of cancer recurrence and depression. Catastrophizing also moderated the predictive effect of invasive rumination on depression. This result can also be explained by the risk enhancement model. Invasive rumination, which causes patients to constantly recall painful cancer experiences, is a negative mental reaction. Moreover, the use of a catastrophizing emotion regulation strategy is “adding insult to injury,” which can aggravate the symptoms of depression.

This study involved patients with cancer during hospitalization and treatment as participants and explored the fear of cancer recurrence and depression as well as the effects of invasive rumination and catastrophizing strategies on fear of cancer recurrence and depression. This provides a feasible reference for clinicians to understand the emotional state of patients with cancer and offer them appropriate psychological counseling.

### Limitations

There are some limitations to this study that need to be further improved in follow-up research. This study used a questionnaire to investigate. Owing to the difficulty of sampling, the number of participants was limited, and there was no detailed classification of the information, such as the type and time of diagnosis of cancer. In future studies, testing in different cities, expanding the sample size, involving patients with specific types of cancer, or adding a control group can be considered to make the research findings more generalizable. Some words in the questionnaire may cause patients to give false answers to seek social approval. In addition, this study only collected information about the psychological state of patients with cancer at a certain time, as it was a cross-sectional study, and did not track the long-term development and changes in the psychological state. Therefore, future research should consider a variety of research methods to explore the relationship between fear of recurrence, invasive rumination, catastrophes, and depression in patients with cancer.

## Conclusion

Invasive rumination plays a mediating role in the relationship between fear of recurrence and depression in patients with cancer. Catastrophizing moderates the relationship between fear of recurrence and depression as well as the relationship between invasive rumination and depression.

## Data availability statement

The raw data supporting the conclusions of this article will be made available by the authors, without undue reservation.

## Ethics statement

The studies involving human participants were reviewed and approved by Ethics committee of Anhui Normal University. The patients/participants provided their written informed consent to participate in this study.

## Author contributions

LQ and QS: conceptualization and project administration. XW and LQ: data curation, methodology, investigation, and writing—original draft. XW, XZ, and JS: resources. LQ, WL, and JS: writing—review and editing. All authors read and agreed to the published version of the manuscript.
